# County Hospital

**Published:** 1866-09

**Authors:** 


### COUNTY HOSPITAL.

The Directors of the County Hospital deserve great credit for the successful and complete organization of the first and only public Charity Hospital of this city. The Institution is not only creditable to the management, but to our city and the entire Northwest, as in all respects it will bear favorable comparison with any similar charity on this continent.

The different departments are now in perfect order, fully equipped, and systematically conducted. The surgical department, as a field for clinical observation and study, is unsurpassed in the West. Since the organization of the Hospital, over twenty capital surgical operations have been performed by the attending surgeons. The lying-in department has also proven eminently successful. Two large wards have been appropriated to this service, and the department affords the very best facilities for the study of this specialty.

The medical department is always replete with every variety of disease. It presents a large field for the study of auscultation and percussion.

An opportunity for studying diseases of the eye is also afforded in the department appropriated to ophthalmic patients.

Recently the Directors have further added to the usefulness and completeness of the Institution by the establishment of an out-door department for the benefit of out-door patients. This is attended daily by the members of the Hospital staff. In addition to this, and to perfect the whole, a large and commodious dead house has been erected, affording good accommodation for post-mortem examinations. A pathological museum, which has already been commenced, contains some very interesting specimens.

We trust a new era has dawned in the medical culture of the Northwest. An opportunity for clinical instruction, for bedside observation and study, to the numerous students of this city, has been a desideratum greatly needed; and now that we have an hospital, so perfect and complete, affording an abundance of material, and every facility for a thorough clinical course, we hope those young men preparing to enter the profession will avail themselves of its advantages.

We will again remind our readers that the attending physicians give four regular clinics a week from October to August, and two a week during August and September. The clinical days are Tuesdays and Fridays, at $1\frac{1}{2}$ o'clock P. M.

